# The social, psychological, emotional morbidity and adjustment techniques for women with anal incontinence following Obstetric Anal Sphincter Injury: use of a word picture to identify a hidden syndrome

**DOI:** 10.1186/s12884-016-1065-y

**Published:** 2016-09-21

**Authors:** M. R. B. Keighley, Yvette Perston, Elissa Bradshaw, Joanne Hayes, D. Margaret Keighley, Sara Webb

**Affiliations:** 1 Institute of Cancer and Genomic Sciences, College of Medical and Dental Sciences, University of Birmingham, Birmingham, UK; 2 University Hospitals Birmingham NHS Foundation Trust, Birmingham, UK; 3 St Marks Hospital London, Harrow, Middlesex, UK; 4 Retired GP, Leeds, UK; 5 Institute of Metabolism and Systems Research, College of Medical and Dental Sciences, University of Birmingham, Birmingham, UK; 6 Delivery Suite, Birmingham Women’s NHS Foundation Trust, Mindlesohn Road, Edgbaston, Birmingham, B15 2TG UK

**Keywords:** Obstetric anal sphincter injury, OASIS, Psychological trauma, Morbidity, Anal incontinence

## Abstract

**Background:**

To identify the emotional, social and psychological consequences and recovery process of anal incontinence (AI) following obstetric anal sphincter injuries (OASIS) and explore if this can be identified as a recognisable syndrome with visual representation.

**Methods:**

A qualitative approach was adopted for this study. Data derived from case studies (*n* = 81) and interviews (*n* = 14) with women with AI after OASIS was used to identify the emotional, social and psychological consequences of AI after OASIS. Keywords and synonyms were extracted and the power of these statements displayed as a ‘word picture’. The validity and authenticity of the word picture was then assessed by: a questionnaire sent to a group of mothers who had experienced this condition (*n* = 16); a focus group attended by mothers (*n* = 14) and supported by health professionals (*n* = 6) and via interviews with health professionals (*n* = 12) who were involved with helping mothers with AI following OASIS.

**Results:**

Women with AI resulting from OASIS have a specific syndrome – the ‘OASIS Syndrome’ - which we have uniquely visualised as a *‘*word picture’*.* They feel unclean which results in dignity loss, psychosexual morbidity, isolation, embarrassment, guilt, fear, grief, feeling low, anxiety, loss of confidence, a feeling of having been mutilated and a compromised role as a mother. Coping relies on repetitive washing (which may become a ritual), planning daily activities around toiletry needs, sharing, family support, employment if possible and attention to the baby. Recovery and healing is through care of the child and hope generated by love within the family.

**Conclusions:**

This study has identified a previously unrecognised ‘OASIS Syndrome’ and, by way of a new and unique ‘word picture’, revealed a hidden condition. There should be greater awareness by the public and profession about the ‘OASIS Syndrome’ and a mechanism for early identification of the condition and referral for management. This, if successful, would overcome the barrier of silence which surrounds this currently unspoken taboo.

## Background

Anal incontinence (AI) in women who sustain obstetric anal sphincter injuries (OASIS) [1], is common. A large Australian study of 1507 nulliparous women reported a 16.6 % faecal incontinence rate 12 months after delivery but confined the definition of incontinence to leakage of solid or liquid stool [2]. When broken down to symptoms at 12 months the same group reported flatus incontinence in 36.2 %, faecal urgency in 21.3 % and faecal leakage in 6.7 % [3]. Very few of the women with these injuries who are faecally incontinent are identified either by their general practitioner or their maternity or child health nurse. This condition rarely resolves over time and the rate of bowel incontinence twelve years later even when flatus incontinence was not included was 14.0 % [4]. As a consequence there is a high rate of bowel incontinence in the community [5] in which primigravida are most at risk [6]. Since incontinence to gas can be just as disabling as other forms of imperfect bowel control, AI has been defined as the involuntary loss of flatus, liquid or solid faeces as well as faecal urgency in this particular clinical setting [7]. These injuries have a devastating impact on the health of the mother and the rest of the family marked by grief, silence and striving for normality [6].

If these injuries are accurately identified and competently repaired at birth, the outcome is generally good, many have no bowel symptoms at all in the short term unless the internal anal sphincter remains defective [8–13]. Unfortunately some of these women have been denied a successful repair or have never had their injury repaired because it was not correctly identified at birth [14, 15]. Some never receive the benefit of being referred for early support, physiotherapy and assessment. Many of those with AI after OASIS are ashamed and feel unable to talk about their symptoms and hide their condition. If these mothers are brave enough to overcome the embarrassment of their condition they may be greeted by a profession that misunderstands or lacks interest and knowledge of how to investigate and provide support for their affliction in which social isolation and psychological morbidity plays a prominent role [2, 8, 16, 17]. Lack of awareness results in less than one in five being asked if they suffer from AI after childbirth [2], despite the known risk factors of first vaginal birth and assisted delivery in particular [18–20]. Consequently many of these women are not recognised as having sustained OASIS until later in life. When these injuries are discovered some mothers explore civil litigation in order to discover what might have gone wrong as well as to fund the care requirements or employment consequences of their injuries.

The aims of this study were to describe the emotional, social and psychological consequences of AI after OASIS, the coping strategies which lead to recovery, and to explore if there is a recognisable syndrome to describe these and construct a ‘word picture’ to visualise the consequences of this syndrome.

## Methods

### Design

**Fig. 1:**
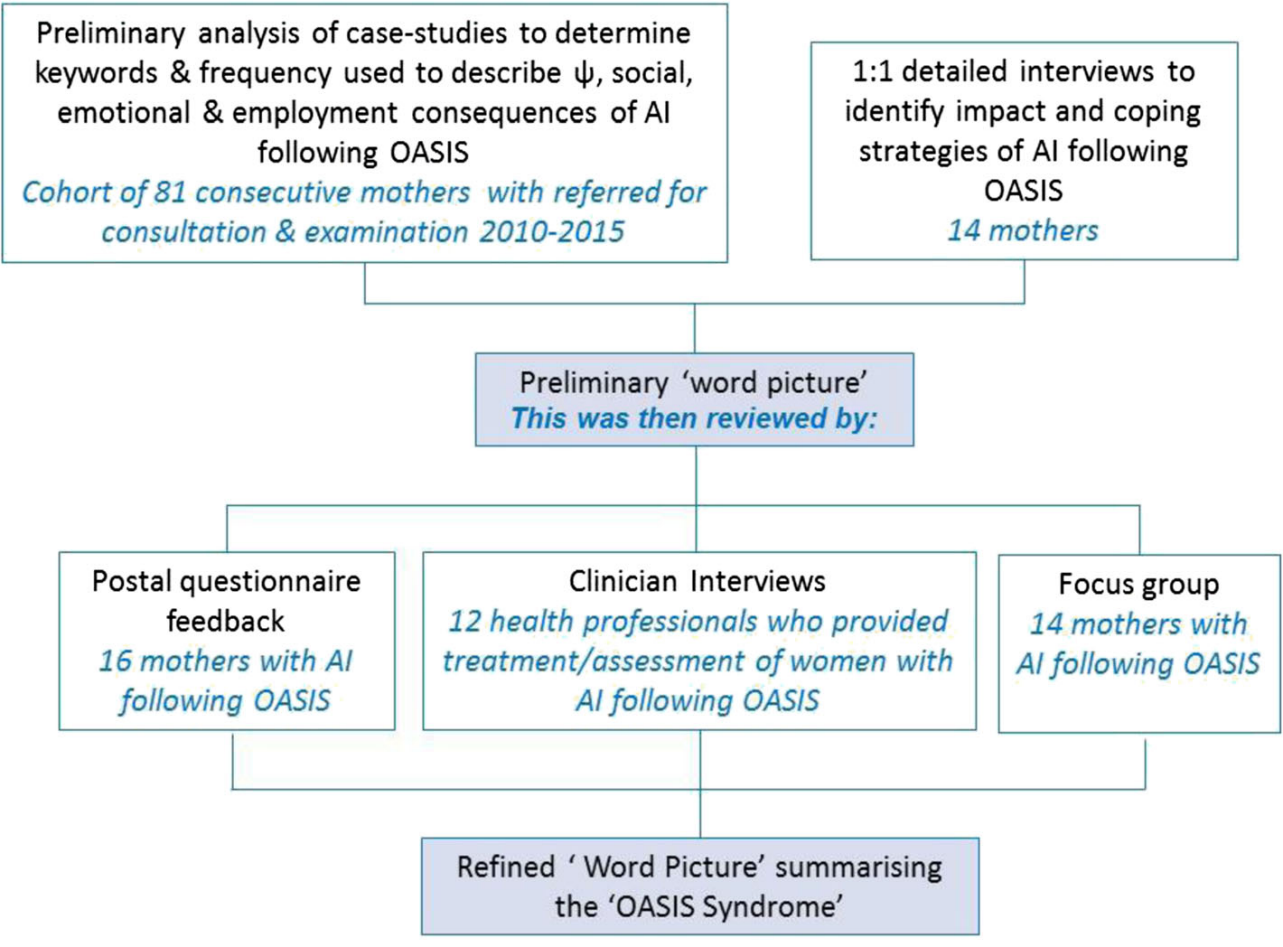
Flow chart to show study structure to formulate the ‘*Word Picture*’

Narrative research was used to explore the social reality of living with the consequences of AI following OASIS by the identification of themes. From data collected from case studies and interviews, keywords or phrases that reflected the lived reality of AI following OASIS were identified and a preliminary ‘word picture’ was developed. The validity and authenticity of the final ‘word picture’ was assessed by a questionnaire sent to mothers with AI following OASIS*,* a focus group and conversations with health professionals involved with helping mothers with the condition (Fig. 1).

### Data collection

#### Initial case studies

This consisted of a cohort of 81 consecutive mothers with AI following OASIS referred to the first author for interview and examination between 2010 and 2014. These interviews were commissioned by lawyers (acting for the claimant or a defendant Trust) who were investigating a possible negligence claim and required a condition and prognosis report. These case studies, which included a full medical history and clinical examination, provided both an assessment of the demographics of OASIS and, as the consultations involved an assessment of the impact of AI on daily living, information about the psychological, emotional and social morbidity of the condition. During consultation quotations were recorded to describe the consequences of the injury, as well as the process of adjustment, coping and recovery of health.

#### Face-to-face interviews

A more detailed series of face to face interviews then took place in 2015 amongst 14 mothers. These interviews were conducted to explore and verify the consequences and coping strategies of AI following OASIS identified in the case study cohort, rather than the demographics of OASIS because medical records were not uniformly available for these women.

#### Focus group

A focus group of 14 women consisted of a mixture of new mothers (*n* = 6) with AI following OASIS and some participants from the case study cohort (*n* = 6) and the face-to-face interview group (*n* = 2). None of these mothers were involved in litigation at the time (four had been involved earlier in cases that had settled). Those attending the focus group were supported by health professionals (*n* = 6) in case there might have been any distress in discussing an event which had been associated with pain, anxiety and dignity loss. Understanding the predicament of these mothers and any adjustment to the ‘word picture’ was explored in depth within the dynamic of the focus group. Furthermore those attending were given the opportunity to modify the preliminary ‘word picture’.

#### Health professionals

Interviews with 12 health professionals involved with the assessment, treatment or support of women with AI following OASIS were undertaken to ascertain their experiences of women undergoing AI following OASIS and to validate the ‘word picture’.

#### Postal questionnaire group

A group of 25 mothers with AI following OASIS, consisting of a mixture of new mothers (*n* = 15) and some participants from the case study cohort (*n* = 10) to whom preliminary ‘word picture’ was sent for assessment and validation. None of these mothers were involved in litigation at the time.

### Analysis

#### Identifying keywords/themes and data saturation

Spontaneous repetitive themes from the case studies and interviews were grouped into words and phrases which provided a theoretical framework for the mothers own living human experiences of AI in OASIS. The words or phrases that satisfied a saturation of themes were identified. Data saturation was defined as a constant comparative process that came to an end when no new categories emerged.

#### Constructing a ‘word picture’

The power, rather than the frequency of the statements identified in the case studies and the face to face interviews was then conveyed by the size of a word or phrase so as to create a ‘preliminary word picture’ to describe the psychological, social and emotional consequences of the condition as well as a pathway toward coping and healing. This methodology stemmed from a word cloud incorporating not only the condition, but also coping and recovery mechanisms. However word cloud methodology only facilitates capture of the frequency of the condition under review and not the ‘force’ (power) and emotional magnitude of the condition. Having captured frequency we then ranked the expressed responses by word size to reflect the ‘force’ of the statements. This resulted in a preliminary *‘*word picture’*.* A colour code was used: black for the condition, green (italics) for coping mechanisms and red for healing/recovery factors. To our knowledge, this technique has not been used before in this format.

#### Testing and refining the ‘word picture’

The preliminary ‘word picture’ was then adjusted and refined by (i) postal questionnaire (Appendix 1) to mothers, (ii) interviewing health professionals who advised mothers with AI from OASIS and by (iii) a focus group. The questionnaire was sent to 25 mothers of whom 16 replied (six returned as wrong address). The questionnaires asked in principal if they agreed with the preliminary ‘word picture’ and whether they wished to modify it in any way by adding or removing words or making existing words or phrases larger or smaller than represented. The interviews with health professionals were used to check the words and phrases used in the preliminary ‘word picture’ without changing the data derived from the mothers. The focus group further contributed to the final ‘word picture’ by reinforcing what had been constructed and adding some fine tuning based on a group dynamic following discussions between mothers who had been injured.

The criteria used for determining the truth of the themes were: credibility by repetitive spontaneous reference to the same themes, transferability particularly in relation to ethnic variables, avoidance of bias by excluding litigation as a cause for certain themes or their emphasis and dependability by testing the responses by interviewing a wide range of health professionals involved with the care of mothers with AI after OASIS. These enquiries identified themes within a specific real life context when the boundaries between the emotional response and the injury were unclear. This provided an understanding of the condition, coping mechanisms and factors that helped to restore the broken sufferer to a repaired state.

### Ethics and consent

Ethical committee approval for the study was granted by the University of Durham as this formed a part of a wider study for a higher degree. There was approval for the use of all data from the women in the case study cohort, for the wording of the information sheet and consent forms, for the interviews both with mothers and health professionals and for the postal questionnaire. Separate ethical approval was obtained for the subsequent focus group. Participants were given the opportunity to decline being interviewed; two of those invited to the face to face interviews did so. Likewise attendance at the focus group was entirely optional and four declined to take part. The focus group attendees were informed that health professionals would be present to explain, support and inform them about the arrangements for the afternoon’s activities and would be present to facilitate the group dialogue and record verbatim statements. Permission to present this study for publication was sought from all participants who were interviewed and all members of the focus group.

## Results

### Demographics of the participants

Participant characteristics from women involved with case studies and face-to-face interviews are shown in Table 1. Detailed demographics for the focus group women, those sent the questionnaire and health professionals were not available.

**Table 1:** Table to show baseline characteristics of participants in the case studies and face-to-face interview groups, values are numbers (%) unless otherwise stated

	Initial case studies	Face-to-face interviews
Number	81	14
Age at index delivery, mean (range) years	34 (17–42)	32 (27–40)
Age at consultation/interview, mean (range) years	38 (21–45)	35 (30–43)
Parity at index delivery		
0 previous births	68 (83.95 %)	11 (78.57 %)
1 previous birth	6 (7.41 %)	2 (14.28 %)
2 previous births	3 (3.7 %)	0
3 previous births	4 (4.94 %)	1 (7.14 %)
Time between consultation & index delivery, mean (range) years	4 (2–11)	3 (1–6)
Mode of index birth		
Spontaneous vaginal birth	24 (30 %)	Data unavailable for these women
Instrumental birth	57 (70 %)	
Mode of instrumental birth		
Ventouse	11 (19.3 %)	
Forceps	23 (40.35 %)	
Ventouse & Forceps	23 (40.35 %)	

Of the cohort of 81 case studies, 50 women lived in the UK of whom eight were Asian (five Muslim, two Hindu and one Sikh) and 31 were from Eire. All 81 women were being assessed for a possible litigation claim.

Of the 14 women participating in face-to-face interviews 10 lived in UK, one was Asian (Muslim) and four were from Eire. Only six were involved with litigation.

The 14 focus group attendees consisted of 13 women from the UK of whom three were Asian (two Muslim and one Hindu) and one was from Eire. None were involved with litigation at the time.

The 12 health professionals consisted of: two colorectal nurses, one midwife, one obstetrician, one involved with public health, one physiotherapist, one gastrointestinal physiologist, one female colorectal surgeon, one psychologist, one psychiatrist and two hospital chaplains one of whom was Muslim. All of the health professionals had first-hand experience of mothers who had suffered AI after OASIS.

The 16 women who responded to the questionnaire consisted of 14 from the UK, two of whom were Asian (one Muslim and one Sikh) and two were from Eire. None were involved with litigation at the time.

### Anatomical trauma and symptoms

The initial cohort of 81 case-studies provided data on actual extent of anatomical damage and symptoms of women with AI after OASIS. This information was unavailable for the other mothers in the study. An anatomical injury was present in 79 (98 %) women (Table 2). Only two women had a neuropathy alone, twenty three had a neuropathy and an anatomical injury. All 25 women with neuropathy had urinary incontinence. There were 41 (54 %) incorrect assessments of the injuries sustained at birth: 25 missed third degree tears and 16 missed fourth degree tears. There were 24 women who sustained either an ano-perineal fistula (*n* = 10) or a form of recto-vaginal fistula (*n* = 14).

**Table 2:** Table to show initial diagnosis of perineal trauma and correct classification of perineal trauma in 79 women participating in case studies (2 of total cohort of 81 women had neuropathy and no tear), values are numbers (%) unless otherwise stated

	Perineal trauma (*N* = 79)	
	Classification at index birth	Classification following detailed examination
Degree of perineal trauma^a^		
1st degree	0	0
2nd degree	25 (31.65 %)	0
3rd degree:	41 (51.89 %)	48 (60.76 %)
3a	4	6
3b	20	18
3c	17	24
4th degree:	13 (16.46 %)	31 (39.24 %)

^a^Classification:

1st degree – Injury to the perineal skin and/or vaginal mucosa

2nd degree – Injury to perineum involving perineal muscles but not involving the anal sphincter

3rd degree – Injury to perineum involving the anal sphincter complex:

Grade 3a tear: Less than 50 % of external anal sphincter (EAS) thickness torn

Grade 3b tear: More than 50 % of EAS thickness torn

Grade 3c tear: Both EAS and internal anal sphincter (IAS) torn

4th degree – Injury to perineum involving the anal sphincter complex (EAS and IAS) and/or anorectal mucosa

Symptoms from the 81 case studies are listed in Table 3 and the consequences of AI following OASIS in this group are presented in Table 4.

**Table 3:** Symptoms in the cohort of 81 mothers with bowel incontinence after childbirth

Bowel incontinence	81 (100 %)
Flatus incontinence	79 (98 %)
Severe urgency (deferment <2mins)	77 (95 %)
Soiling	73 (90 %)
Impaired rectal evacuation	65 (81 %)
Urge incontinence	64 (79 %)
Passive incontinence	28 (35 %)
Urinary incontinence	42 (52 %)
Sexual dysfunction	79 (98 %)
Marital distress	70 (86 %)
Intercourse no longer spontaneous	61 (75 %)
Intercourse no longer orgasmic	52 (64 %)
Fear of faecal leakage during intercourse	52 (64 %)
Painful during intercourse	40 (49 %)
Never resumed intercourse	10 (12 %)
Failed partnership/marriage (at time of study)	7 (8 %)

**Table 4:** Consequences of faecal incontinence after childbirth

Anxiety	80 (99 %)
Social restriction	80 (99 %)
Embarrassment about flatus incontinence	78 (96 %)
Leisure compromise	77 (95 %)
Feeling unclean	77 (95 %)
Difficulty coping	75 (93 %)
Travelling difficulty	73 (90 %)
Feeling ashamed	68 (84 %)
Loss of dignity	68 (84 %)
Feeling degraded	68 (84 %)
Leakage of waste during exercise	68 (84 %)
Loss of confidence	68 (84 %)
Compromised motherhood	60 (74 %)
Feeling low	49 (60 %)
Feeling isolated	43 (54 %)
Anxiety about having another baby	40 (49 %)
Antidepressant medication	31 (37 %)
Fear of leaving the house because of incontinence	24 (30 %)
Anger	23 (28 %)

Data from Case Studies *n* = 81

### Themes describing the impact of AI following OASIS – recognition of the ‘OASIS Syndrome’

Narrative research identified the following themes: cleanliness, ritual, repetitive processes, feelings derived from the consequences of injury, self-worth, emotional sequelae, non-disclosure of events and feelings, motherhood issues, adjustment to/coping with the condition as well as healing and moving forwards. The themes and their frequency in the 14 interviews have been tabulated and ranked in frequency (Table 5).

**Table 5:**
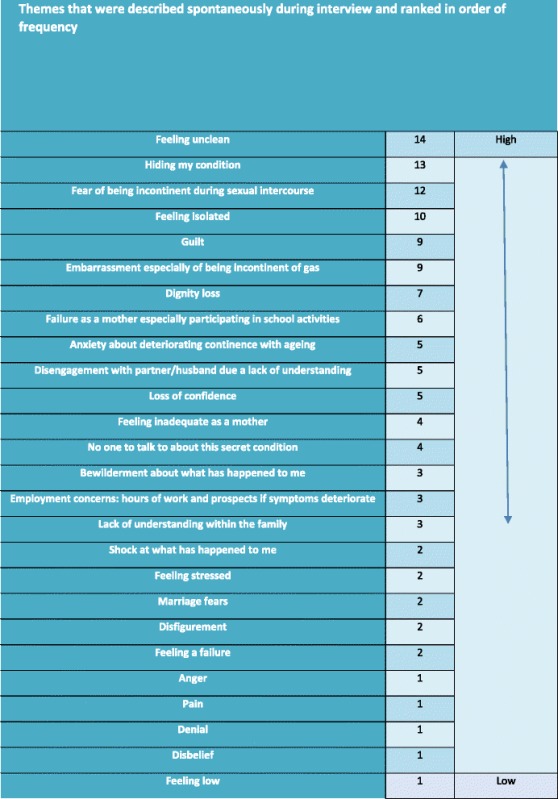
Themes that were described spontaneously during interview and ranked in order of frequency

Data from one to one interviews *n* = 14

#### The condition

The dominant themes which were repeatedly expressed in the case studies (CS), interviews (I) and focus group (FG) are described, in order of importance. These themes are illustrated by individual quotations of anonymised statements from participants. These statements are specific and some are infrequent but highlight severity whilst others are more general and common. The source of these individual statements is provided using interviewee reference number in each category: CS, I and FG.

A dominant feature was the hidden taboo where mothers described hiding their condition for a variety of reasons such as shame, bad memories, feeling injured and dignity loss leading to social isolation which some expressed by becoming a prisoner in their own home and feeling alone:

*‘I feel too ashamed to talk about these things’* (I,5); *‘I prefer to keep it a secret’ (*CS 14*); ‘I am afraid to go out of the house*’ (CS 18); *‘I feel isolated’* (CS 35); *’There was no one I could talk to’* (CS 61).

Mothers felt unclean and expressed this in a variety of ways which impacted on their sense of wellbeing, dignity and in some cases their religious practices:

*‘I feel constantly unclean’* (CS 24; I 3) *‘I am obsessed with washing’* (FG 3); *‘I do not feel clean enough to pray’* (FG 10).

There were expressions of a loss of dignity, feeling a failure and being diminished which lead to anxiety and becoming emotionally fragile:

*‘I feel a failure’* (CS 33); *‘Apart from being very anxious, stressed and emotionally drained, I find my condition degrading’* (FG 5).

There were profound expressions of specific psychosexual morbidity. Uniformly prominent was a constant fear of being incontinent during sexual intercourse, which had a negative impact on their relationship with their spouse. Some mothers amongst our immigrant community found it particularly difficult to discuss these issues with their spouses and with health professionals:

*‘He is hardly a husband anymore; he is more like a carer’* (CS 6); *‘We have not had any sexual encounters since the birth over 3 years ago’* (CS 54); *‘It is never spontaneous because I have to shower beforehand in case I might be dirty’* (CS 69).

The embarrassment of these new symptoms was frequently expressed during interview:

*‘I have no control of wind which is a great embarrassment’* (CS 4).

Some mothers felt guilty about their condition in which they also felt a failure:

*‘I blame my baby’* (CS 72); *‘I feel I have failed everyone’* (FG 2).

There was anxiety generated by painful memories:

*‘I have flashbacks of what has happened at her birth’* (I 3).

There was a fear of becoming incontinent when they were no longer in their own home:

*‘I have panic attacks especially when I go out of the house’* (CS 27).

Some mothers felt depressed and emotionally labile:

*‘I am often tearful’* (I 8); *’My condition has made me very anxious.*’ (CS 66).

There were comments about being injured and no longer feeling attractive and some mothers used the word mutilated:

*‘I do not feel attractive; it strips away your femininity’* (I 2).

Frequently mothers expressed feelings of a loss of confidence:

*‘I need a lot of support and reassurance’* (CS 44).

Many mothers talked about compromised motherhood particularly in relation to school activities:

*‘I often feel bad that I cannot join in with all the school activities….. I leak if I take vigorous exercise with the children’* (CS 62).

Many expressed concern that there was a lack of information that this condition can happen to many mothers. Some mothers said that if they had been warned beforehand that this might occur they would have been less likely to have blamed the baby for their condition:

*‘Nobody warned me about this’* (FG 6).

The consequences of work compromise were expressed by many who had planned to return to the workplace:

*‘I have lost my job’* (CS 35); *‘I have had to change my position at work…..I leak if I have to lift heavy objects’* (CS 44).

#### Coping strategies

The most frequent spontaneously expressed coping mechanism was to repetitively wash in order to feel clean, which for some had become an obsession. In this regard the Asian practice of using a hose for washing the perineum was adopted by some Caucasian mothers who found thereby that skin excoriation and the time spent on the toilet was much reduced. Rectal washout although very invasive was successfully practiced by some mothers:

*‘I have to wash very frequently in order to feel clean’* (CS 22); *‘I have become obsessed about showering’* (I 3); *‘I must know where the toilets are located’* (CS 64).

The other dominant coping mechanism was needing to plan the day and being aware of and having access to toilet facilities:

*‘I have to plan everything’* (CS 46); *‘I have to carry a change of clothing and washing things whenever I leave the house’* (I 8).

There was a variable expression of wanting to share their condition with others. Some wished to hide their symptoms whereas others found it helpful to talk to family and close friends who were understanding and sympathetic:

*‘I have been very fortunate to have found someone I can talk to* (CS 4); *‘My family have been very supportive’* (CS 36).

Adjustment by simply focussing on daily tasks was expressed by some mothers:

*‘I find that trying to get on with life is the best solution to my physical problem’* (CS 80).

For those who had been able to return to work, many, despite the need to adjust, found that having a job took their mind away from their all-consuming condition:

*‘I am grateful that I have a job, it has helped me’* (CS 66).

#### Factors in the process of recovery and healing

Despite the fact that having a baby had been responsible for their condition, most mothers identified the baby as being the pivotal focus for recovery:

*‘Although my problem was all the result of the birth…having him and feeding him was a way of taking my mind off the stress’* (CS 63).

An existential aspect to healing and recovery that involved overcoming physical and emotional pain, moving on from a state of anger to one of forgiveness, becoming positive rather than remaining in a state of despair and which was encapsulated in a sense of hope was expressed by women in different ways:

*‘There has been an existential component to the recovery process’* (FG 2); *‘I feel I have an inner strength that is hard to put into words’* (CS 49); *‘I have not given up hope’* (I 7); *‘I am encouraged by knowing that there are many other mothers with this condition that have learnt to cope’* (FG 2).

Although the condition placed a strain on relationships, those whose spouse had provided support considered that this had been an important aspect to the road to recovery. Also those who found someone they could talk to and share with during therapy gained a person who facilitated the healing process:

*‘My loving husband has been a brick’* (FG 11); *‘We really helped each other through group therapy’* (FG 1).

Recovery, often a slow process, was enhanced by support from others especially within the family by love, blessings of everyday life, the gift of the child and maintaining a sense of humour:

*‘It is nice to be able to laugh about it’* (FG 8); *‘I keep reminding myself of the gift of my child’* (FG 11); *‘My family are very supportive’* (FG 3).

### A ‘word picture’ depicting the ‘OASIS Syndrome’

**Fig. 2:**
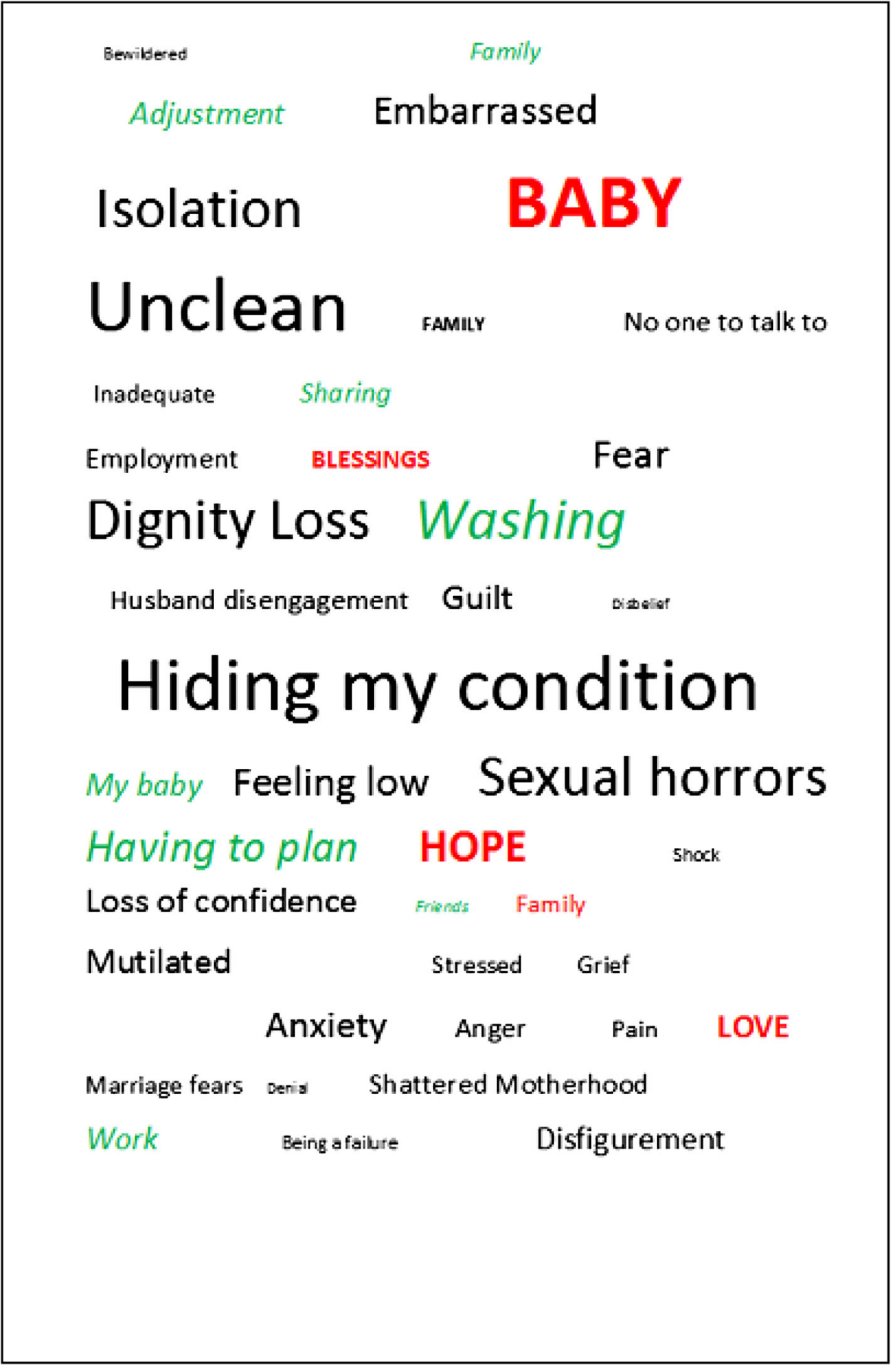
Word picture

The result of the 16 replies to the questionnaire on the preliminary ‘word picture’ indicated that only nine women suggested alteration, seven of which related to changes in emphasis of some of the words, whilst the remaining two suggested additions or deletions. The health professionals did not wish to modify what had been agreed by the mothers but reinforced that the picture graphically described the emotional response to the injuries in the mothers they had counselled or treated. The focus group felt that some of the words needed minor modification and corrected a spelling mistake. The final ‘word picture’ is presented in Fig. 2. The authors believe that this describes, in a novel, graphic form, a specific syndrome which we term the ‘OASIS Syndrome’ of the social, psychological and emotional consequence and recovery process of women who suffer from AI following OASIS.

## Discussion

Women with AI resulting from OASIS have a specific syndrome – the OASIS Syndrome - which we have described in a *‘*word picture’ in which the size of the words represents the emotional force of expression. They feel unclean which results in dignity loss, psychosexual morbidity, isolation, embarrassment, guilt, fear, grief, feeling low, anxiety, loss of confidence, a feeling of having been mutilated and a compromised role as a mother. Coping relies on repetitive washing (which may become a ritual), planning daily activities around toiletry needs, sharing, family support, employment if possible and attention to the baby. Recovery and healing is through care of the child and hope generated by love within the family. The OASIS Syndrome is largely unrecognised by the profession because it is hidden by the sufferer on account of the associated dignity loss and sexual morbidity.

There are few studies that specifically identify the social, psychosexual and emotional consequences of AI after OASIS. Most studies provide a general overview of the impact of AI on quality of life [21–27] but unlike this study few specifically acknowledge the union of these consequences we have now recognised as the ‘OASIS Syndrome’.

A Danish study interviewing nine mothers with AI in OASIS emphasised conflict as a prominent feature whereby mothers were described as ‘being engaged in an *“everlasting fight”* to keep up an appearance of being a healthy person’ [22]. Interestingly, this is in contrast to the findings from our study where most mothers suffered in silence, few were angry and a sense of conflict was often fuelled by exploring the litigation process.

Findings from our study on the negative impact that AI following OASIS has on body image, sexual function and anxiety is supported by others [8, 28]. One common theme from our study and others is that mothers with AI following OASIS all lack professional support, knowledge of the condition, information and coping strategies for themselves and their partners [8, 22, 28], despite this deficit in professional clinical practice and service provision having been highlighted over 15 years ago [29].

We believe our study highlights some critical implications to health care providers. The OASIS Syndrome, which affects at least one in ten of all mothers having a baby [30], remains largely unknown by the public or the medical professions. There are well documented risk factors for these injuries [18] the two most important and most easily identified are first vaginal delivery and assisted birth. It has been previously reported that, even in these high risk groups, anal sphincter injuries remain common. With correct identification and immediate repair following the birth these women have a good prospect of avoiding the consequences of this syndrome [9, 11]. Unfortunately, the findings from our study clearly demonstrate that many of these injuries are commonly missed or misclassified [14].

There are now compelling arguments for informing mothers about the risk of vaginal delivery and of OASIS in particular [31], to enable women with imperfect continence prompt referral for early investigation, assessment, support and treatment [29]. If a mother has an identified injury which is repaired at birth then they should have the benefit of a multidisciplinary postnatal clinic so that early advice and support can be provided long term but unfortunately this is not universally available in the UK [32]. The bigger problem however is the undetected injury in an uninformed mother who discovers that she is anally incontinent and does not know whether this is a normal consequence of having a baby and is too embarrassed to talk to the profession and seek help because she feels stigmatised. Even if she has the courage to make an appointment with her general practitioner there is a high chance that she will not be listened to, assumed to be a sufferer of irritable bowel syndrome and not identified as a person who needs specific referral to a focussed facility for identification of the injury and support [2].

Findings from discussions during the focus group highlighted bowel function as an area of physiology that did not warrant attention in the postnatal period from health professionals. Consequently it was suggested by more than one mother that this deficit could be remedied by a simple mechanism for identifying mothers with bowel incontinence by using a nationally accepted set of questions about bowel symptoms. We are now exploring an appropriately worded questionnaire to identify AI following OASIS which would allow access to a comprehensive care pathway for those with AI, an idea that has already been independently suggested by others [33]. Such a care pathway could empower mothers who have been injured themselves, but whose condition has improved as a result of advice and treatment, to act as a ‘wounded healer’ for the recently diagnosed sufferer [34]. The ‘wounded healer’ would give support and encouragement, and, provided she was appropriately selected, could reinforce hope, reconciliation and focus through reciprocity which could prove to be a powerful tool in the healing process, particularly in the process of restored cleanliness from this unspoken taboo [35]. Likewise discovering a ‘buddy’ during the early stages of therapy could be beneficial especially the opportunity to share the complexities of adjustment within the family. An alternative would be to establish support groups throughout the country where mothers with the condition could meet one another.

### Strengths and implications

The *‘*word picture’ is a novel method of encapsulating a syndrome and emphasising its dominant components. It has not been developed before. It provides an understanding of a complex social, psychological, emotional consequences and recovery factors of AI following childbirth. The *‘*word picture’ would serve as a simple aide memoire to provide timely, multidisciplinary support for these mothers. The ‘word picture’ might also be used as a tool to evaluate the severity of the ‘OASIS syndrome’ as well as the impact of therapy, support or a change in healthcare policy on the wellbeing of the mother who has AI from OASIS by allowing the mother the opportunity to modify the picture via word deletion, addition, enlargement or diminishment.

The case study data were potentially a biased sample since many had severe injuries and were exploring civil litigation because they believed that there may have been a mistake or substandard care. However, this bias was rectified by interviews and a focus group with non-litigants.

It is not clear from this study whether the described syndrome is applicable to older women at a different stage in the OASIS Syndrome journey. Some mothers have little or no symptoms after vaginal delivery despite extensive sphincter injuries and are only later referred to pelvic floor clinics. Surprisingly this may not occur until sometime after the menopause or during aging. Consequently the *‘*word picture’ may not be applicable to mothers who present later in life.

## Conclusion

This study has identified a previously unrecognised ‘OASIS Syndrome’ and, by way of a new and unique ‘word picture’, revealed a hidden condition in which women with AI following OASIS are dominated by feelings of being unclean, resulting in dignity loss, social isolation, guilt, loss of sexual intimacy, a negative impact on motherhood and feminine identity. There should be greater awareness by the public and profession about the ‘OASIS Syndrome’ and a mechanism for early identification of the condition so that multidisciplinary treatment and support can be provided in a national agreed care pathway. This, if successful, would overcome the barrier of silence which surrounds this currently unspoken taboo.

## Appendix

**Questionnaire to mothers**

**Background:**

Prof. Keighley has created a coloured picture of words to describe the situation that he has found amongst 90 women that he has spoken to over the last five years who suffer from bowel incontinence after childbirth.

The picture is an average position as he sees it. Clearly everybody is different. Some find some issues a much bigger hurdle than others.

The choice of words is based on the words used by mothers during these interviews.

The size of the words represents the relative importance of some matters over others. The size is not based on calculation from numerical data because these emotions are very difficult to quantify. The size is based on the relative importance/emphasis and frequency of spontaneous reporting during interview by this one assessor.

**Feedback and validation:**

In order to obtain some feedback, the plan is to send the coloured word picture to two groups:

- A group of the 90 mothers who provided the original information (MRBK’s patients seen for reports)
- A group of other women who suffer from bowel incontinence seen and treated within the first five years of the start of symptoms (We will approach patients being seen and treated by Ellie Bradshaw and Yvette Perston)

What we ask mothers to do who are willing to help with Feedback/validation:

**Question1**. Does this coloured picture, which represents the bad things in Black, the things that keep you going in Red and the coping strategies in Green paint a picture that represents roughly what you have been through?............Yes? No? (delete what does not apply)

**Question2**. As we are all different please mark the picture in a way that describes you best. To do this (1) place a line through words which do not apply; (2) add words that you feel have been missed out; (3) put a square round words that you think should be given greater emphasis; (4) put a circle round the words that should be given less emphasis.

**Question3**. Which group of mothers do you belong?:

(1) Prof Keighley; (2) Ellie Bradshaw; (3) Yvette Perston? (delete what does not apply)

Thank you.

Please return in the stamped addressed envelope but do not give your name, it is important that this is anonymous.

Professor M.R.B. Keighley.

## Abbreviations

AI: Anal incontinence
CS: Case studies
FG: Focus group
I: Interviews
OASIS: Obstetric Anal Sphincter Injury
